# The Fight against Disease

**Published:** 1921-04-30

**Authors:** 


### THE FIGHT AGAINST DISEASE.

We welcome the appearance of a first number of the new form of Reports by the Research Defence Society. It is hoped that they will now be published regularly six times a year by Macmillans, at a price of 6d. Instead of to members and associates only, they will in future be sent to all the chief public libraries, and obtainable through any bookseller or newsagent. The aim of the Society is to provide considered extracts and reviews of recent research work in a form intelligible to the lay public.

In this way it appears that a dual purpose will be served. Nowadays the general reader eagerly absorbs whatever medical information his press provides for him. But unfortunately it is as yet impossible to say that this press publishes the really instructive matter. Also, although the day of serious opposition to the rights of experimental medicine is rapidly waning, yet there is urgent need of financial support, and this will only be forthcoming when there is general appreciation of the facts.

The key-note of the latest issue is the greatness of victories in prevention rather than treatment of disease. This is no new theme, but at the same time it is one which bears considerable repetition, and is particularly well instanced in tuberculosis. Lieut.-Col. Nathan Raw has in this country provided one of the most recent and hopeful suggestions on preventive inoculation, and this—not a "cure"—was the essential import of his paper before the Royal Society of Medicine. The more we set ourselves to think of tuberculosis in man and in cattle, the more we long for the discovery of a *protective* treatment, such as we have against typhoid. And we may well believe that Calmette, also, at the Pasteur Institute has recently brought us nearer to that discovery. He has succeeded in attenuating the germs of bovine tuberculosis, passing them through many generations in the process. By inoculation with this weakened virus it is probable that other animals can be protected against the disease at its natural strength.

There is also an interesting sketch given of the St. Mary's and Middlesex exhibits at Olympia, but these we have already reviewed, and their value amply justifies the Defence Society's suggestion to the Health Ministry. "Here is a grand opportunity for the Ministry of Health. Why should it not start some real objective teaching? The British public is fed-up with printed teaching. Incessant tracts, reports, monographs, blue-books, notifications, newspaper articles—these are poured out by the million, in tons. Germs are talked to it every day and all day long. But they are never shown to it."

There is a good deal in this suggestion, although we cannot see it materialising at present. The greatest of modern health questions is how to help the public to help themselves. Many methods are available, and in its particular efforts we wish the Research Defence Society well.

One must not forget, however, one of the most amusing sections of this report, which awards the first Nonsense Prize to a gentleman in Manchester who is reported to have publicly stated—in all solemnity to urge antivivisection—that "there is an apparatus on sale for the boiling and baking of animals whilst alive, and for recording the varying intensity of their cries before death!" There are second and third nonsense prizes, but, as illustration, one will amply suffice.

